# Sequence-based calculation of local energetic frustration in proteins

**DOI:** 10.1063/4.0000781

**Published:** 2025-11-24

**Authors:** Adam M. Kuhn, Vinícius G. Contessoto, José N. Onuchic, George N. Phillips

**Affiliations:** 1 Department of Physics and Astronomy, Rice University, Houston, Texas 77005, USA; 2 Department of Chemistry, Rice University, Houston, Texas 77005, USA; 3 Department of Biosciences, Rice University, Houston, Texas 77005, USA; 4 Center for Theoretical Biological Physics, Rice University, Houston, Texas 77005, USA

## Abstract

Given proteins' fundamental importance in human health and catalysis, the relationships between protein sequence, structure, dynamics, and function have become a topic of great interest. One way to extract information from proteins is to compute the local energetic frustration of their native state. Traditionally, energetic frustration calculations require protein structures as a starting point. However, using a single protein structure to evaluate the energetic frustration for a given amino acid sequence does not always fully represent the protein's structural ensemble. Therefore, we have developed a sequence-based method to evaluate energetic frustration in proteins using direct coupling analysis and statistical potentials. Our approach exhibits significant agreement with established structure-based frustration methods in terms of their mutual agreement with crystallographic B-factor. Moreover, our sequence-based method shows elevated precision in classifying high B-factor residues, suggesting that it has some robustness to unstructured regions of proteins.

## I. INTRODUCTION

**FIG. 1.**
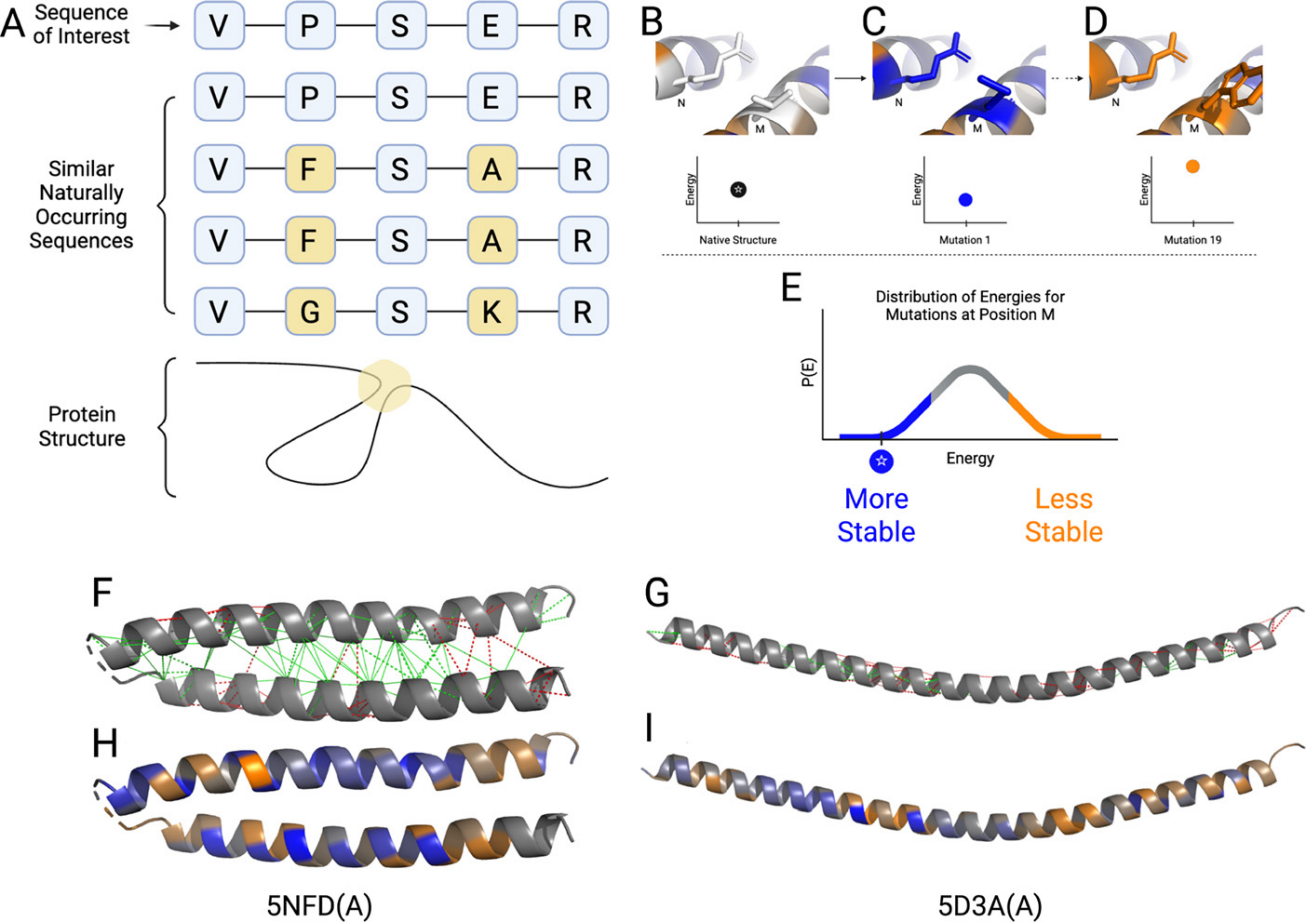
Energetic frustration analysis, shortcomings, and alternatives. (a) The degree of covariance between different positions along the polymer chain can be measured by coupling scores, where larger coupling scores indicate stronger covariance. Thus, theoretically, coupling scores can be used instead of structure for calculating the energy of a protein provided there exists a reliable known interaction energy for nearby amino acids (statistical potentials).^9^ (b) The native interaction between amino acids N and M represents one possible energetic state. (c) and (d) Single amino acid mutations at position M create new interaction energies that may be more (blue) or less (orange) energetically favorable. (e) The distribution of all possible mutation energies at position M reveals whether the native sequence represents an optimized interaction (blue region) or a frustrated contact (orange region). An important caveat is that the same sequence of amino acids can adopt different conformational states [(f)/(h) and (g)/(i)], leading to distinct frustration patterns in structure-based analysis. Panels are labeled with the corresponding PDB ID and chain identifier to indicate the specific structural models depicted. (f) and (g) Green lines represent minimally frustrated contacts, while red lines represent highly frustrated contacts between amino acid pairs. (h) and (i) The two-dimensional contact-based frustration patterns can be compressed into one-dimensional, residue-level frustration maps, where blue regions indicate minimal frustration and orange regions indicate high frustration.

Proteins, strings of amino acids, are the primary functional units of all known life.^1^ Upon synthesis, they reliably fold into complex three-dimensional structures necessary for their biological function.^2^ In recent years, considerable progress has been made in predicting these three-dimensional protein structures. AlphaFold, ESMFold, and RosettaFold are just a few examples that have revolutionized structural biology through large-scale protein structure prediction.^3–6^ Interestingly, not all protein structure prediction tools are trained on protein structure data. Instead, they operate implicitly on the principle of coevolution, which allows one to infer the proximity between amino acids based on the observed covariance of amino acid identities at different positions in a multiple sequence alignment (MSA). This method, often called direct coupling analysis (DCA), follows from the observation that amino acids near one another in a protein's folded structure are likely to be rendered more fit if a deleterious substitution mutation is accompanied by a compensatory substitution nearby.^7^ AlphaFold's success, and the success of others who pioneered this method,^7,8^ is a testament to the validity of coevolutionary reasoning [Fig. 1(a)].

Although advances in protein structure prediction are valuable and exciting, structures rarely tell a protein's full story. Other tools from simulation biophysics, experimental molecular biology, and bioinformatics are still essential for comprehensively understanding the connections between a protein's sequence, structure, dynamics, and function. One useful lens for protein inquiry is energetic frustration analysis. Energetic frustration is defined as the phenomenon that occurs when a system exhibits mutually exclusive local and global energy minima. As a result, some regions of the system are forced to assume suboptimal energetic configurations while the system is in its global energetic ground state. Due to the large number of inter-amino acid interactions, some degree of energetic frustration is exceedingly common in proteins.^10^ Regions of high mutational frustration are often functionally relevant sites. These regions can be binding sites for small-molecule substrates, other proteins, or allosteric regulators, or they can play a more subtle role in the functional mechanism of catalytic and motor proteins.^11,12^ Thus, localizing energetic frustration in proteins is an essential capability for understanding the functional mechanisms of proteins and their interactions.^13^ Furthermore, frustration calculations are computationally inexpensive, making them convenient and scalable. Traditionally, regions of high and low energetic frustration are identified using a protein's structure.^14,15^ From this structure, the distances and forces between all of the amino acids can be calculated. Then, substitution mutations are introduced to create “decoys.” By comparing the energetic stability of the native protein with that of the decoys, one can infer the relative stability of different contacts in a protein. This information can be further compressed into a scalar representation of the relative energetic stability of each native amino acid [Figs. 1(b)–1(e)].

Current tools like the Frustratometer have had great success in identifying local frustration in proteins by applying the mutational method described above to static structures.^14^ However, the use of structure-based methods produces a structure-dependent frustration output. While this can be valuable for analyzing the difference in frustration patterns for different protein configurations, it can also be a source of ambiguity when searching for functionally relevant energetic frustration [Figs. 1(f)–1(i)]. As a result, a complementary method that looks at frustration from a different perspective may be valuable.

To remedy the structure dependence of current frustration analysis methods, we propose a new sequence-based method for localizing energetic frustration in proteins. This method capitalizes on the biophysical theory of coevolution and statistical (knowledge-based) potentials for amino acid interactions to generate a map of a protein's mutational frustration.^9^ Theoretically, this approach has a number of possible benefits. By using evolutionary coupling scores as a substitute for inter-residue distance, additional information regarding the biological significance of each pair of amino acids is baked into the output. Specifically, it is possible for two amino acids to be near one another in real space but not exhibit significant coevolution (particularly in unstructured regions of a protein). These “false contacts” can contribute to a misleading signal when evaluating energetic frustration from a protein structure. Thus, using evolutionary coupling scores in place of inter-residue distances provides a filter for contacts that have been deemed functionally necessary by generations of natural selection.

For benchmarking our new sequence-based score, referred to herein as sequence-based frustration, it was tested against traditional structure-based methods on 20 randomly selected high-quality monomeric proteins and 20 highly flexible proteins. An additional comparison was needed in instances of disagreement between the two local frustration scores, for which we employed crystallographic B-factors—a measure of atomic position uncertainty in x-ray crystallography—under the assumption that thermal motion will correlate with the degree to which energetic interactions are optimized.^16^ We observed that the contour of the two local frustration signals correlated with one another in the randomly selected monomeric proteins, suggesting that the substitutions enabling sequence-based frustration calculations are generally reasonable. Notably, however, the correlation between sequence-based frustration and crystallographic B-factor was more reliable than the correlation between structure-based local frustration and B-factor for the set of dynamic proteins. This may be an indication that the filtering done implicitly when calculating coupling scores preserves a local frustration signal that different members of the protein's structural ensemble have in common. Additionally, sequence-based frustration and structure-based local frustration show complementary strength in classifying residues with high and low B-factor, respectively.

## II. RESULTS

### A. Sequence-based frustration agrees with structure-based methods in randomly selected high-resolution monomeric protein crystal structures

**FIG. 2.**
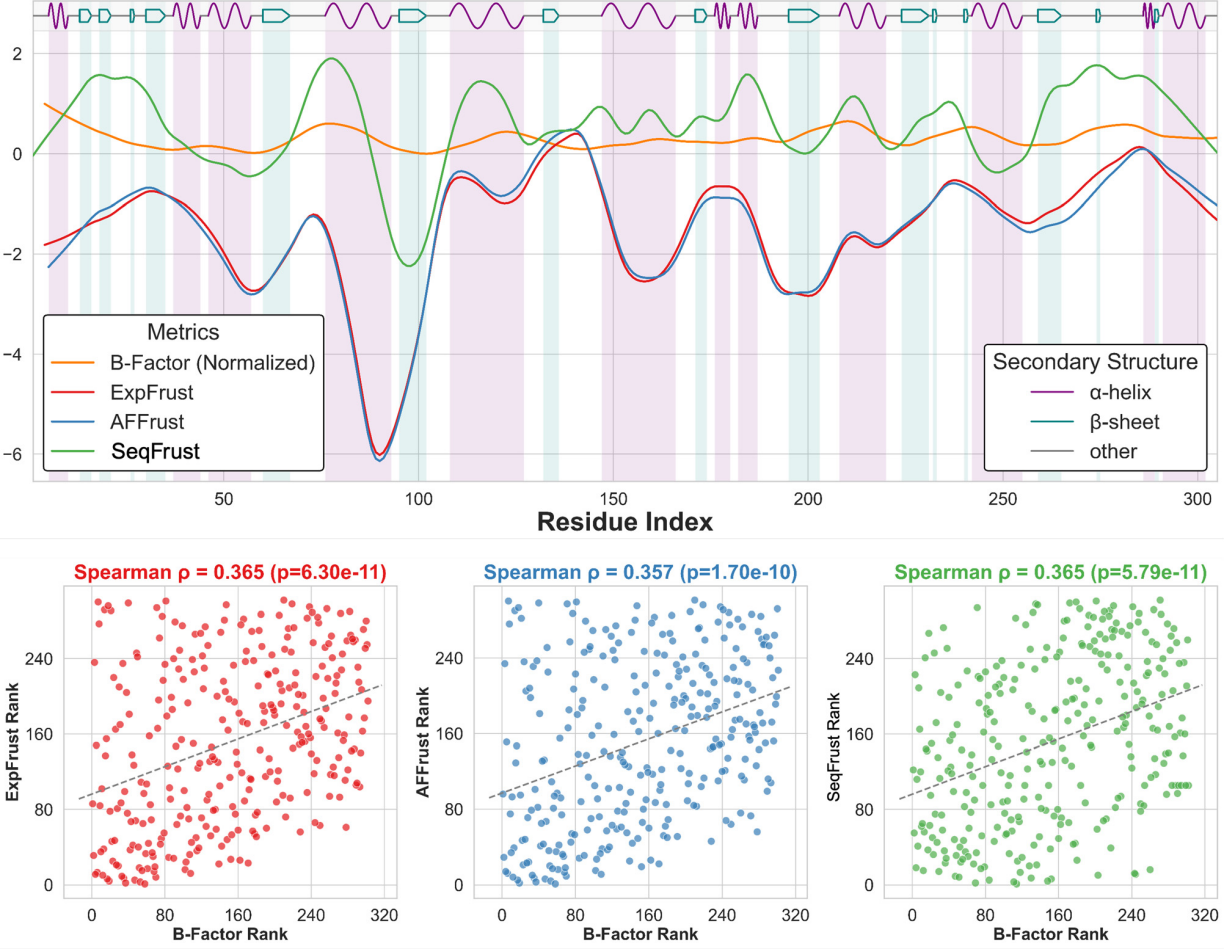
Example analysis of local frustration accuracy (PDB ID: 1QI0). Top: Smoothed local frustration profiles and B-factors across residues. Bottom: Rank correlations between B-factors and each local frustration score. Three approaches are compared: Experimental frustration (ExpFrust, red) uses experimentally determined structures, AlphaFold Frustration (AFFrust, blue) uses predicted structures, and sequence-based frustration (SeqFrust, green) uses evolutionary coupling scores instead of structure. Note: Crystallographic B-factors reflect not only thermal motion but also static disorder, crystal packing effects, and experimental noise.^17^ Such factors may reduce the strength of the observed relationship.

**FIG. 3.**
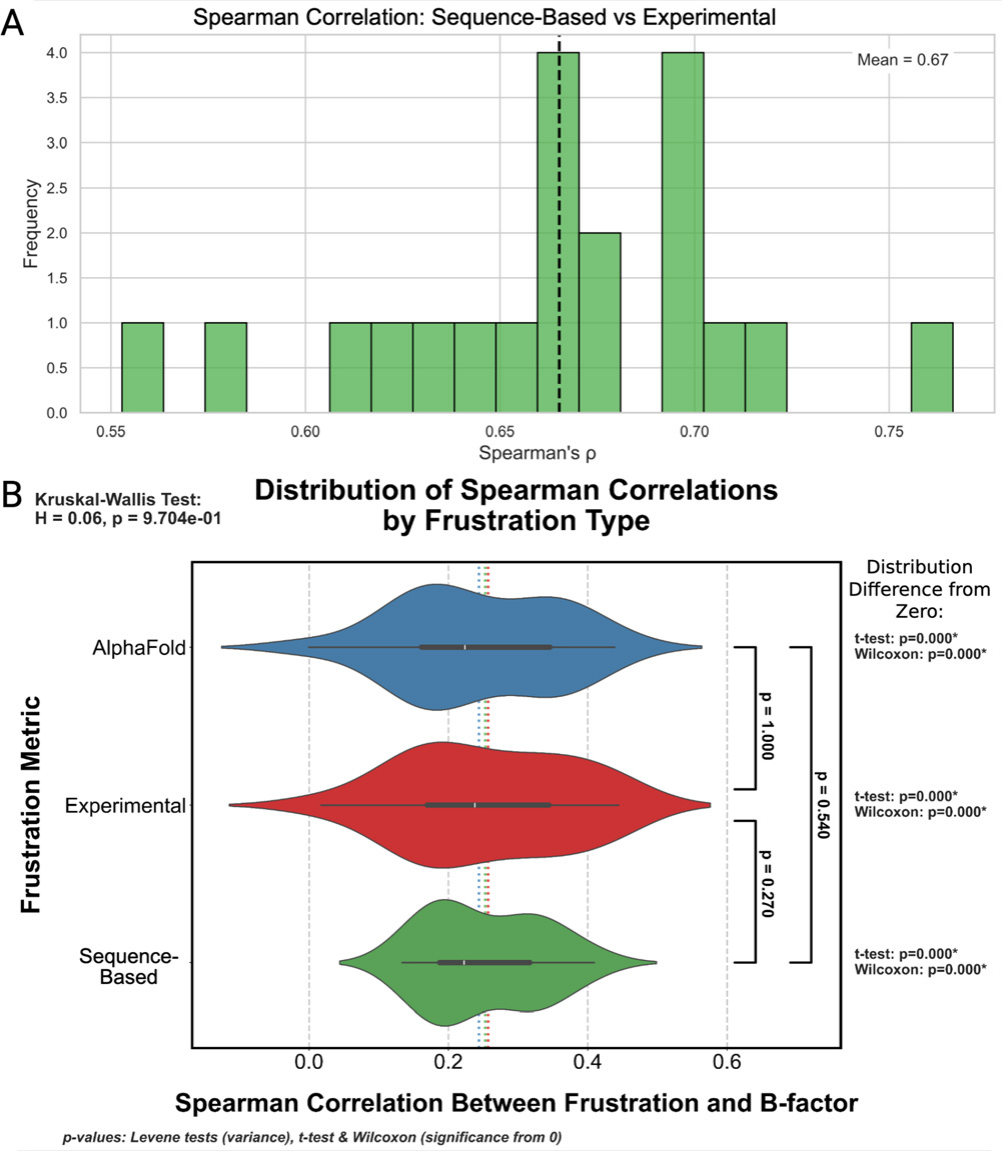
(a) Spearman correlation between evolutionary and experimental frustration across proteins. Histogram of Spearman correlation coefficients between residue-level evolutionary and experimental frustration for 20 high-resolution ( ≤ 3 Å) monomeric proteins. Values closer to 1 indicate strong agreement in the rank order of local frustration across residues. A vertical dashed line marks the mean Spearman ρ across all proteins. Sequence-based frustration was calculated using coevolutionary coupling scores and statistical potentials, while experimental frustration was derived from structure-based mutation energy analysis. (b) Comparison of the distributions of Spearman correlation coefficients between each local frustration score and B-factor for 20 high-resolution ( ≤ 3 Å) monomeric proteins. Three approaches are compared: Experimental frustration (ExpFrust, red) uses experimentally determined structures, AlphaFold Frustration (AFFrust, blue) uses predicted structures, and sequence-based frustration (SeqFrust, green) uses evolutionary coupling scores instead of structure. Median and quartiles are shown by the white tick and box plot within each violin. Means are represented by the colored dashed lines in the background.

After developing the framework for sequence-based frustration with a test set of proteins, its efficacy was cross-validated using a randomly selected group of high-quality ( ≤ 3 Å resolution) monomeric crystal structures. The agreement between the three scores of interest, sequence-based frustration, structure-based local frustration, and B-factor, was evaluated using Spearman correlation coefficients, in which the covariance of the rank of the scores is assessed. A sample analysis for endoglucanase Cel5A from *Bacillus Agaradhaerens* (PDB ID: 1QI0) is shown in Fig. 2.^18^ The figure shows the smoothed local frustration profiles from our sequence-based method (green) and the Frustratometer output from both an experimental crystal structure (red) and the corresponding AlphaFold predicted structure (blue). Moreover, the smoothed normalized experimental B-factor (gold) and secondary structure are displayed. Finally, below the main plot are three subplots showing the rank correlation between each local frustration score and the experimental B-factor. Above each plot is the Spearman correlation coefficient between the two variables and the p-value for the hypothesis test for the significance of the Spearman correlation coefficient, showing that each correlation is indeed significantly greater than zero. Together, these plots illustrate the manner in which each protein's local frustration profile was evaluated. It should be noted that the local frustration signal has been smoothed to give the representations in Fig. 2. Raw local frustration signal can be viewed for all proteins in the 20R dataset at evolutionaryfrustration.com. Figure 3(a) shows the Spearman correlation coefficients between evolutionary and experimental (structure-based) frustration. The motivation for using rank correlation is that there is no clear evidence to assume that structure-based and sequence-based local energetic frustrations are linearly correlated, given that the energies were calculated in very different ways. However, raw local frustration data and Pearson correlation coefficients are shown in the supplementary material (Fig. S41).

The Spearman correlation coefficients between each local frustration type and crystallographic B-factor for the set of 20 high-resolution monomeric proteins are summarized in Fig. 3(b). Structure-based local frustration analysis was performed on AlphaFold predicted structures to test the degree to which coevolutionary filtering influenced the observed local frustration signal in these structures. T-tests and Wilcoxon signed rank tests show that the distributions of Spearman correlation coefficients are significantly greater than zero, indicating that a weak but systematic monotonic relationship exists between local frustration and B-factor. Kruskal–Wallis and Bonferroni-corrected pairwise Levene's tests reveal that the medians and variances of the distributions of Spearman correlation coefficients between local frustration and B-factor do not differ significantly. This suggests that our sequence-based method exhibits a monotonic relationship with experimental B-factor that is as reliable as that observed for established structure-based methods.

### B. Sequence-based frustration identifies highly frustrated residues with greater precision than structure-based methods in randomly selected high-resolution monomeric protein crystal structures

Crystallographic B-factors reflect not only the thermal motion of atoms in a protein crystal but also static disorder, crystal packing effects, and experimental noise.^17^ Although Spearman correlation is sufficient to establish the existence of a monotonic relationship between energetic local frustration and B-factor, it cannot directly assess the discriminative power or robustness to noise of a particular frustration score. Thus, to better evaluate the accuracy of frustration scores, we employed classification-based methods such as receiver operating characteristic (ROC) and precision-recall (PR) analysis. ROC-PR analysis identifies how well a continuous score predicts a binary classifier at various thresholds.

**FIG. 4.**
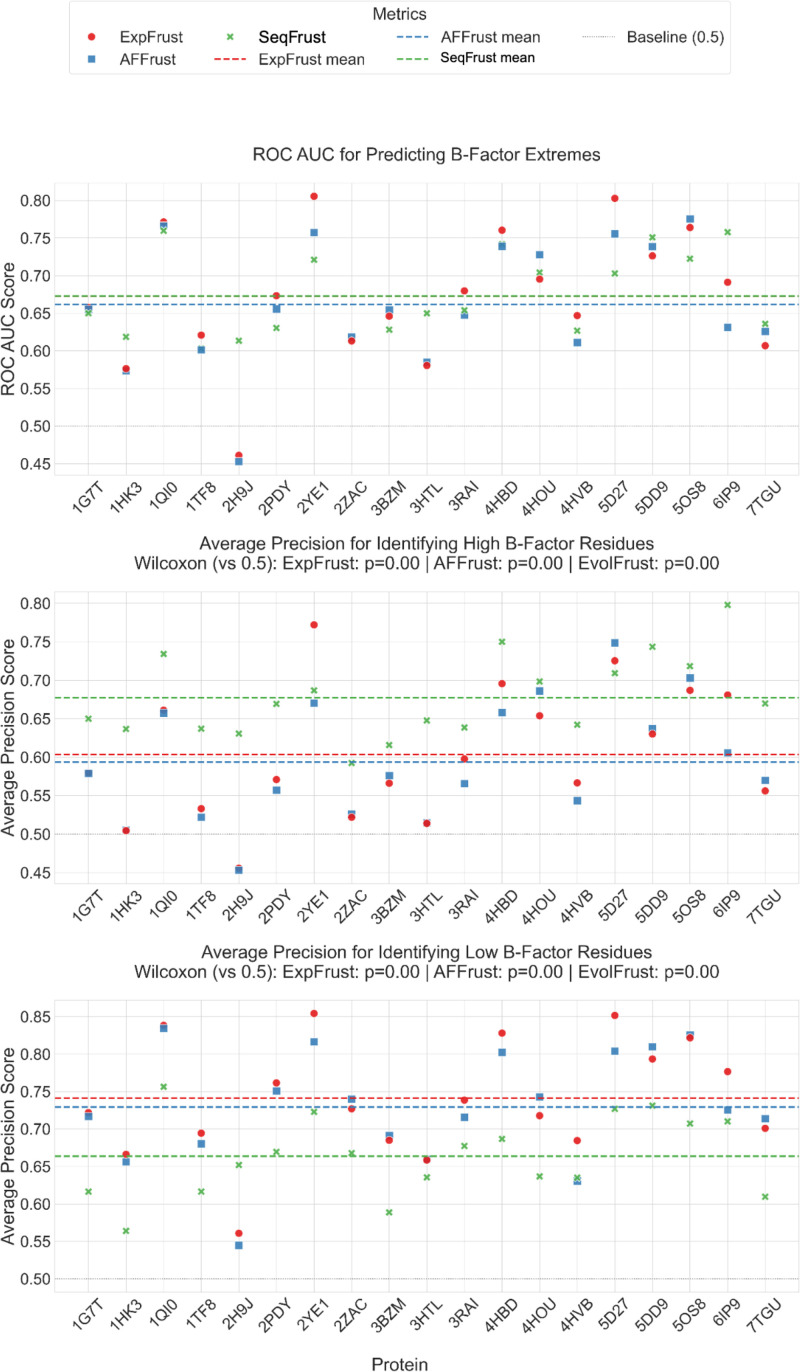
Accuracy of different frustration scores in predicting B-factor extremes. (Top) Area under curve (AUC) for receiver operator characteristic (ROC) curves and (middle, bottom) average precision recall (PR) for 20 randomly selected high-quality ( ≤ 3 Å resolution) monomeric proteins. AUC-ROC values greater than 0.5 indicate that true positive predictions are more likely than false positive predictions. Values of average precision greater than 0.5 indicate that the frustration score correctly identifies high (middle) or low (bottom) B-factor residues more often than it incorrectly labels (middle) low B-factor residues as high or (bottom) high B-factor residues as low. The Wilcoxon signed rank test was used to determine if the distribution of average precisions for each frustration score is significantly greater than baseline. P-values are shown above (middle) and (bottom).

For the purposes of these classification-based tests, residues in the top and bottom quartiles of each protein's B-factor distribution were treated as the ground truth for highly and minimally frustrated residues, respectively. Then, for each frustration score and each protein, the threshold for classifying a residue as highly or minimally frustrated was increased systematically, and for each threshold value, the accuracy of the frustration score was assessed. The results of this procedure can be found for each protein in supplementary material Figs. S1–S20, while the aggregate results are summarized in Fig. 4. We observed that sequence-based frustration identifies high and low B-factor residues with precision significantly greater than random chance. Additionally, sequence-based frustration identified high B-factor residues with greater precision than structure-based methods and low B-factor residues with lower precision, suggesting that the two methods may have complementary strengths.

### C. Sequence-based frustration exhibits better agreement with B-factor than structure-based mutational frustration in dynamic proteins

**FIG. 5.**
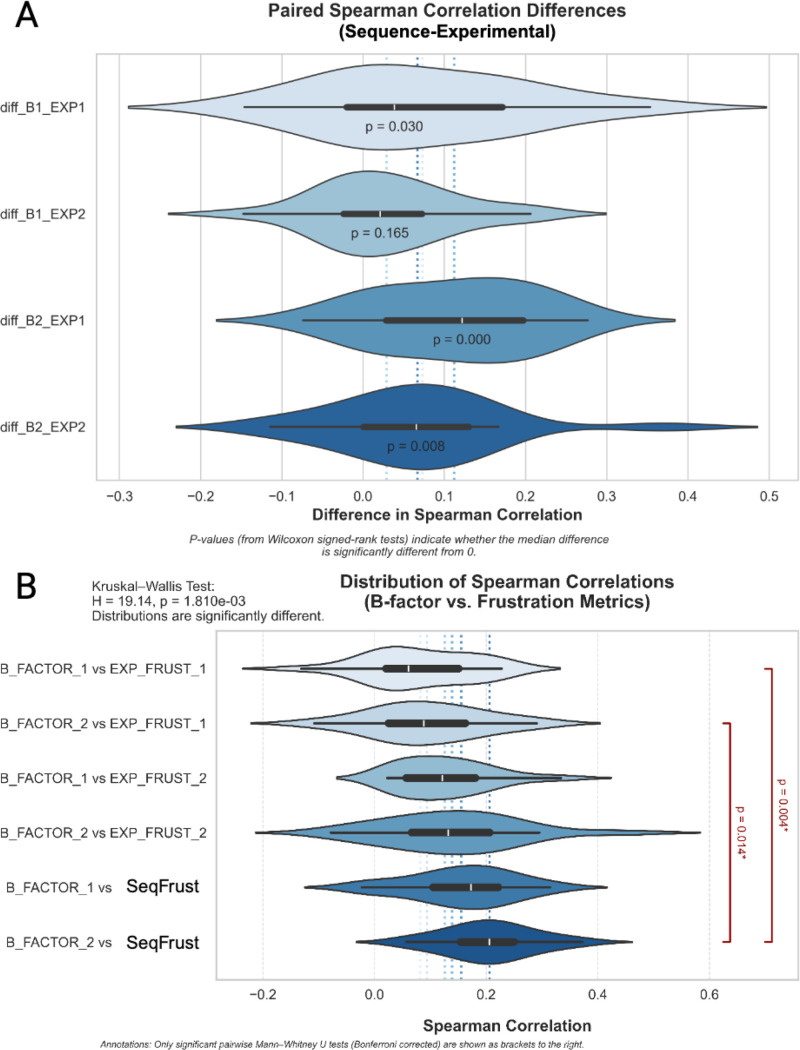
(a) Paired analysis of frustration-B-factor Spearman correlation coefficients in a set of 20 dynamic proteins. For each of the 20 sequences analyzed, two protein structures were identified. Spearman correlation coefficients were calculated between each frustration type (sequence-based, experimental structure 1, experimental structure 2) and the B-factor from each of the experimentally determined protein structures, generating 6 coefficients per protein. Summarized above are the distributions of the differences between the sequence-based frustration-B-factor Spearman coefficients and the experimental frustration-B-factor Spearman coefficients. Positive values indicate a stronger monotonic relationship between sequence-based frustration and B-factor than between experimental frustration and B-factor. (b) Spearman correlation coefficients between frustration and B-factor for proteins with exceptional conformational variability. For each protein sequence analyzed, two experimentally observed structures were identified and used for comparison. Shown above is the distribution of Spearman correlation coefficients between each of the frustration types (sequence-based, experimental structure 1, experimental structure 2) and the B-factors from each of the two structures. Distribution medians and quartiles are shown via the box plots within each violin. Means are represented by the color-coded dashed lines in the background of the figure.

Next, we evaluated the difference in performance between sequence-based frustration and structure-based frustration analysis in a set of 20 highly flexible proteins identified via PDBFlex.^19^ See Sec. IV for more information on the curation of this dataset. Figure 5(a) shows that sequence-based frustration regularly exhibits greater agreement with experimental B-factor than does its structure-based counterpart. Furthermore, sequence-based frustration tends to exhibit better agreement with experimental B-factor than structure-based methods, even when the structural frustration and B-factor come from the same protein crystal structure. For completeness, Fig. 5(b) shows the distribution of Spearman correlation coefficients between each frustration type and each structure's B-factor prior to the pairwise comparison done in Fig. 5(a). The significance of each individual Spearman correlation coefficient, as determined by comparison to the approximate null distribution for a large sample size (in this case, the number of amino acids), can be viewed in supplementary material Sec. 6.2 (Figs. S21–S40).

## III. DISCUSSION AND CONCLUSIONS

Energetic frustration analysis is one way to extract functional and dynamic information about a protein given its structure. Sequence-based frustration analysis, our sequence-based method predicated on sequence coevolution, exhibits a number of potential benefits that suggest it could be a meaningful addition to the current suite of analytical techniques for proteins. Sequence-based frustration works by substituting the amino acid contact map from a protein structure with evolutionary coupling scores, which measure the degree of covariance between sequence positions in a multiple sequence alignment (MSA). These coupling scores closely correspond with structural contacts in many cases, but have some key differences. One potential benefit of sequence-based frustration is that it largely excludes contributions from amino acids with functionally irrelevant chemical identities. This filtering effect stems from sequence-based frustration's reliance on coevolutionary coupling scores, which are unlikely to be significant in unstructured regions of proteins. As a result, sequence-based frustration signals are likely to be biased toward structurally relevant residues, making it easy to interpret directly. This is corroborated by sequence-based frustration's ability to identify high B-factor residues with greater precision than structure-based frustration analysis. Thus, sequence-based frustration could be particularly useful for selecting regions of interest for enzyme optimization or allosteric regulatory site identification.

However, sequence-based frustration is likely blind to transient conformations that may be observed in some protein structures. If such a conformation were not an experimental artifact, structure-based frustration methods could provide information that would otherwise be inaccessible. Moreover, structure-based methods appear to exhibit a greater ability to accurately identify minimally frustrated residues.

Another key consideration in frustration analysis is data availability. In the past decade, protein structures have become increasingly easy to access through database curation, neural network prediction, and cloud computing. In contrast, precomputed coupling score resources are much less common. This places the burden of coupling score generation on the prospective user. Coupling score calculations are not prohibitively resource-intensive for small sets of proteins, but it can be challenging to scale up significantly. Nevertheless, it may be possible to reduce the cost of coupling score generation significantly by training a neural network to infer coupling scores from multiple sequence alignments (MSAs) or sequences directly. Similarly, it may be possible to generate synthetic MSAs based on patterns observed in genuine MSAs to reduce computational cost and eliminate blind spots in existing protein sequence data. Once coupling scores or a protein structure is obtained, however, the calculation of frustration is computationally trivial.

## IV. METHODS

### A. Multiple sequence alignment generation

The multiple sequence alignment (MSA) for each protein was generated by using the EVcouplings^20^ (version 0.2) align function against the UniRef100^21^ database of non-redundant protein sequences using the “standard” EVcouplings protocol and bit score thresholding (clustering threshold = 0.8, domain threshold = 0.5, sequence threshold = 0.5). The EVcouplings align functionality utilizes JackHMMR as the backbone of the MSA generation. HMMER^22^ version 3.4 was used for MSA generation herein. The number of jackhammer iterations used for MSA generation was 5. The MSA was filtered such that sequences with less than 50% alignment to the query sequence were removed, and columns for which more than 30% of the entries were gaps (i.e., no residue present) were ignored. This method is distinct from how Pfam MSAs are generated, where many high variability sequences are aligned to one another. With this method, a database (uniref100 in this case) is searched for similar sequences which are then aligned creating a very high-quality MSA.

### B. Coupling score calculation

Coupling scores were calculated by plmc using the standard EVcouplings protocol with 100 iterations.^23^ Gaps in the input MSA were ignored. The strength of regularization of both coupling parameters and fields used was 0.01.

### C. Sequence-based frustration calculation

Sequence-based frustration, Γ, is a sequence and statistical potential-derived scalar designed to represent the energetic frustration of any given amino acid in a particular protein sequence. Two matrices are needed for its calculation: **C** the n × n matrix of coupling scores between each location along the protein chain, and **U** the n × n matrix of interaction potentials where the i,j^th^ entry is the statistical potential associated with the amino acids located at positions i and j. We used Miyazawa–Jernigan potentials for **U**. Upon taking the Hadamard product of **C** and **U**, one obtains

F≡C⊙U,

where F is an n × n matrix where each entry $f_{ij}$ is the interaction potential between amino acids i and j weighted by the MSA-inferred probability that residues i and j are near one another in real space. Summing over all non-redundant $f_{ij}$ gives an estimate of the total energy of the protein. This procedure can be repeated for all possible single amino acid mutants for a given protein sequence with minimal computational effort. The result is a predicted energy for the native protein and all possible single amino acid permutations, which can then be compared to infer the native frustration of each residue. More rigorously,

$$\gamma (\sigma )\equiv \sum_{i<j}f_{i,j}(\sigma ),$$

where σ is the sequence of interest. Then,

$$\Delta \gamma_{m}\equiv \gamma (\sigma^{wt})-\gamma_{m}(\sigma^{\text{mut}})$$

is the energy difference between a given single amino acid substitution mutant, m, and the wild type (wt) protein. Finally, averaging over all $\Delta \gamma_{m}$ for a given location, k, in the protein sequence gives the scalar representation of the frustration for that location,

$$\Gamma_{k}\equiv \sum_{m=1}^{19}\Delta \gamma_{m}/19.$$

It should be mentioned that this method of calculating energetic frustration is distinct from the Z-score-based method used by established tools like the Frustratometer. The goal of this method is to output a scalar for each position that represents whether mutations at a specific location in the protein's sequence tend to increase or decrease the total energy of the folded protein. It does not take into account how the energies are distributed. This would, however, be an interesting topic for future work, given that distribution width likely measures how strongly a given position contributes to the overall energy of the folded structure.

### D. Frustratometer output compression

The Frustratometer output for mutational frustration contains a list of amino acid contacts along with the interaction energy, θ, of each contact and the mean interaction energy calculated for the mutational decoys, ϕ.^14,15^ For simplicity, these data were compressed into a single scalar representation of frustration for each residue. This was done by first collecting all h of the contacts containing amino acid n. Let this set of contacts be called $X_{n}$. For the *m*th element of $X_{n}$,

$$\nu_{m}=\theta_{m}-\phi_{m}$$

was calculated. Finally,

$$\epsilon_{n}\equiv \sum_{m=1}^{h}\nu_{m}/h$$

was calculated and used as the compressed frustration score for the *n*th amino acid. We adopted this method to compress the Frustratometer outputs because it matched how we had calculated and compressed the sequence-based frustration output, thus making for an apt comparison. Our sequence-based method calculates the average energy difference upon mutation. The way in which we compressed the matrix of experimental frustration values is also the average energy difference resulting from mutation. In this way, the two manifestations of frustration are the same.

### E. Experimental data curation

The set of 20 randomly selected high-quality monomer crystal structures was generated by first filtering the RCSB PDB^24^ by x-ray crystallography entries with a resolution of 3 Å or less and containing only protein entities. From the resulting list of 84 354 PDB IDs, 20 were selected at random and used for analysis.

The set of 20 highly dynamic proteins was obtained using PDBFlex.^19^ PDBFlex entries were sorted by max RMSD between cluster members. The entries with the greatest RMSD that were meaningfully represented by the UniRef100 database were used for investigation. The average B-factor for each residue was calculated by averaging over the B-factors of all of the atoms in a given residue.

All PDB identification numbers and PDBFlex cluster identification numbers, along with CATH classifications, can be found in the supplementary material (Tables S1 and S2).

### F. Statistical analysis

All data analyses were performed in a Jupyter Notebook^25^ using NumPy,^26^ pandas,^27^ Biopython,^28^ SciPy,^29^ and scikit-learn.^30^ Plots were generated using Plotly,^31^ Seaborn,^32^ and Matplotlib.^33^ Monomers were extracted from x-ray diffraction-determined biological assemblies stored in the RCSB PDB using PyMol.^34^

## SUPPLEMENTARY MATERIAL

See the supplementary material for more details, including a comprehensive analysis of the local energetic frustration for each protein, both sequence-based and structure-based, as well as the PDB IDs and crystallographic resolutions for each protein, and an evaluation of the Pearson correlation between structure-based frustration and sequence-based frustration.

## Data Availability

The code developed for calculating sequence-based frustration and generating the figures in this text are openly available via GitHub at https://github.com/AKuhn100/EvolutionaryFrustration. Structural and frustration data can be interactively visualized for the set of 20 randomly selected high-quality monomeric proteins in Evolutionary Frustration at https://evolutionaryfrustration.com/dataviz/. All protein structural data used for this work are readily available via the RCSB Protein Data Bank.^24^ The data that support the findings of this study are openly available in the RCSB Protein Data Bank at https://www.rcsb.org/. A full list of PDB accession codes used in this study is provided in the supplementary material (Tables S1 and S2).
